# Postpartum mental health after Hurricane Katrina: A cohort study

**DOI:** 10.1186/1471-2393-9-21

**Published:** 2009-06-08

**Authors:** Emily W Harville, Xu Xiong, Gabriella Pridjian, Karen Elkind-Hirsch, Pierre Buekens

**Affiliations:** 1 Department of Epidemiology, Tulane University School of Public Health and Tropical Medicine, New Orleans, LA, USA; 2 Department of Obstetrics/Gynecology, Tulane University School of Medicine, New Orleans, LA, USA; 3 Woman's Hospital Research Institute, Baton Rouge, LA, USA

## Abstract

**Background:**

Natural disaster is often a cause of psychopathology, and women are vulnerable to post-traumatic stress disorder (PTSD) and depression. Depression is also common after a woman gives birth. However, no research has addressed postpartum women's mental health after natural disaster.

**Methods:**

Interviews were conducted in 2006–2007 with women who had been pregnant during or shortly after Hurricane Katrina. 292 New Orleans and Baton Rouge women were interviewed at delivery and 2 months postpartum. Depression was assessed using the Edinburgh Depression Scale and PTSD using the Post-Traumatic Stress Checklist. Women were asked about their experience of the hurricane with questions addressing threat, illness, loss, and damage. Chi-square tests and log-binomial/Poisson models were used to calculate associations and relative risks (RR).

**Results:**

Black women and women with less education were more likely to have had a serious experience of the hurricane. 18% of the sample met the criteria for depression and 13% for PTSD at two months postpartum. Feeling that one's life was in danger was associated with depression and PTSD, as were injury to a family member and severe impact on property. Overall, two or more severe experiences of the storm was associated with an increased risk for both depression (relative risk (RR) 1.77, 95% confidence interval (CI) 1.08–2.89) and PTSD (RR 3.68, 95% CI 1.80–7.52).

**Conclusion:**

Postpartum women who experience natural disaster severely are at increased risk for mental health problems, but overall rates of depression and PTSD do not seem to be higher than in studies of the general population.

## Background

Disaster increases community psychopathology [1], with depression and PTSD being especially common [1,2]. In most cases, symptoms recede with time, and many victims prove resilient [3]. However, a certain proportion of the population will develop long-lasting problems [4]. Some aspects of the disaster, such as number of lives lost and whether it was natural or human-caused, may enhance psychopathology [1]. The person's own experience of the hurricane also influences their later mental health. Studies have shown that fearing one's life was at risk [4-7], having a relative die [8,9], and being injured [4] all predict psychopathology.

Personal characteristics also affect the risk of mental health consequences. Women are more vulnerable to disaster-related psychopathology than men [6,10-14]. Married men appear to be at lower risk, while married women may be at higher risk [15]. Those of low income or education are also generally at higher risk [6,9,12]. New mothers, who frequently fall into several of these high-risk categories, may be especially vulnerable to the consequences of disasters [15].

Even under the best of circumstances, women may be particularly susceptible to mental health disorders postpartum. Up to 25% of postpartum women experience depressive symptoms [16,17]. The Pregnancy Risk and Monitoring System found that 23.6% of Louisiana women reported being moderately to very depressed after birth, but only 3% had gotten help for this depression [18]. Mothers who are low-income or single are at greater risk [19], as are women who experience significant life events postpartum [20]. Other mental disorders are less studied, but postpartum anxiety, for example, often appears comorbid with, or even more commonly than, depression [21].

Chang et al. reported that 29% of pregnant women had minor psychiatric morbidity after an earthquake [22], and Adams found that mothers of infants who were evacuated due to Chernobyl were at higher risk for distress than controls 11 years later [23]. However, we are unaware of any research that has specifically targeted postpartum mental health in the aftermath of a disaster. We examined the influence of Hurricane Katrina on mental health in a group of postpartum women from southern Louisiana.

## Methods

Hurricane Katrina struck the United States Gulf Coast on August 29, 2005. Participants were recruited from two hospitals, chosen to represent the New Orleans area (Tulane Lakeside Hospital) and a less-affected comparison group (Women's Hospital) in Baton Rouge, LA between March 2006 and May 2007. All women admitted for childbirth were eligible and were approached sequentially. Twenty-three percent of the women were pregnant during Hurricane Katrina. Women recruited at the New Orleans site needed to have lived in the New Orleans area before Katrina, be 18 or over, speak English, and have access to a telephone. Women in Baton Rouge needed to be 18 or over, speak English, have access to a telephone, and not have had a severe exposure to Katrina (defined as being forced to evacuate or having a relative die). Demographic characteristics were similar between the two sites, except that Baton Rouge women were somewhat more likely to be married or living with a partner (87% vs. 78% in New Orleans) and to have a college education or higher (47% vs. 33% in New Orleans).

During their hospital visit participants completed a recruitment questionnaire and completed a phone interview at 8–10 weeks postpartum. 365 women were recruited and 292 (80%) participated in the phone interview. Follow-up continued at six months and one year postpartum, but there was substantial attrition (60% completion), so these data are not presented. However, results were very similar (data available from the author).

All protocols used in the study were approved by the Institutional Review Boards of Tulane University and Woman's Hospital and all patients provided informed consent.

Hurricane experience was based on answers to 9 questions, including whether participants ever felt their life was in danger during the storm, if they or a family member became ill or injured as a result of the storm, if they walked through floodwaters, severity of damage to their home and possessions, if anyone close to them died, or if they witnessed anyone die. The items ask about threat, injury, and loss, which have been shown to be associated with mental health in previous disaster studies [8,11,24]. The scale was based on a previous study of Hurricane Andrew by Kaniasty and Norris [25]. Based on a factor analysis, we created three categories of hurricane experience: damage (at least some damage to home, property, or others' property); illness/injury (to self, household member, or other); and danger (felt life in danger, walked through floodwater, saw someone die). We also created a scale of overall severity of hurricane experience by summing the number of events experienced; this approach has been used in several disaster studies [5,11,24,26,27]. Hurricane experience was measured at recruitment.

The Edinburgh Postnatal Depression Scale (EPDS), a ten-item questionnaire, was used to assess postpartum depressive symptoms among the study participants. Validation studies in general and high-risk populations put sensitivity between 65 and 100% and specificity between 49 and 100% [28,29]. The EPDS has been shown to be valid in non-postpartum populations as well [30,31]. A score of 13 or greater was used as a cutoff, which has been indicated for to correlate well with serious postpartum depression [32].

PTSD was measured using the PTSD checklist (PCL), a commonly used, 17-item inventory of PTSD-like symptoms, with response alternatives ranging from 1 (*not at all*) to 5 (*extremely *[33,34]. PTSD was defined as a score of 3 or more on one reexperiencing, three avoidance, and two hyperarousal criteria. This conforms to the psychiatric definition of PTSD and has been used in other studies [35]. PTSD was also examined as scoring above 50, a cut-off that has performed well against clinical PTSD diagnosis [33,34]. Results were similar and only data from the first definition are included.

### Statistical Analysis

Cross-tabulations and chi-square tests were used to examine the relationships among hurricane experience, demographic characteristics, and mental health outcomes. Log-linear/poisson regression [36] was used to model relative risks with control for potential confounders: age, race, income, education, marital/partnership status, and parity. Time since hurricane was not associated with mental health at 8 weeks. Interaction terms were used to examine effect modification by race, pregnancy status during the hurricane, and hospital of recruitment (New Orleans/Baton Rouge).

## Results

The study population was predominantly white or black, in their mid- to late-twenties, with some higher education (table 1). More of the lower-income, black, or younger women were lost to follow-up. The majority of the women had a moderate experience of the hurricane: most (51%) had some property damage and knew others who were seriously affected (72%); many felt their lives were in danger during the storm (35%). A large majority (87%) of the New Orleans area women evacuated.

**Table 1:** Description of the Katrina Moms study population, southern Louisiana, 2006–2007.

	Initial study population (n = 365)		completed mental health assessment at 8 weeks (n = 292)	
		
	N	%	N	%
Age				
18–22	58	16	44	15
>22–28	116	32	87	30
>28–33	103	28	84	29
>33	88	24	77	26
				
Race				
white	232	65	195	68
black	114	32	83	29
other	13	4	10	4
				
Education				
< high school	37	10	21	7
high school diploma	79	22	60	21
some college/associates' degree	104	29	85	30
college degree	92	26	80	28
> college	43	12	37	13
				
Marital status				
married	214	59	189	65
living with partner	76	21	56	19
separated/divorced	11	3	8	3
never married	60	17	37	13
				
Income				
<$20000	88	25	61	21
$20000–$60000	164	46	136	48
>$60000	101	29	88	31
				
Hospital of recruitment				
New Orleans	249	68	206	71
Baton Rouge	116	32	86	29

* N may not add to column head due to missing data.

Black women were more likely to have damage and to have perceived or experienced danger during the hurricane (data not shown). Multiparous women and higher-income were more likely to have had severe damage. The most educated group was the least likely to have had damage or experienced danger. These relationships remained after mutual adjustment for age, partnership status, parity, race, income, and education. Black women (adjusted RR 1.34, 0.96–1.86) and women with less than a high school education (aRR 3.16 (1.56–6.40) were more likely to have 2 or more severe experiences of the hurricane.

Race was a strong predictor of mental health (table 2), with black women more likely to suffer from depression and PTSD. Women with a high school diploma were most vulnerable to psychopathology, as were women who did not live with a partner. Among the New Orleans area women, multiparous women were more likely than primiparous women to be depressed and have PTSD, but this did not hold for Baton Rouge women.

**Table 2:** Social and demographic predictors of mental health in a cohort of postpartum women exposed to Hurricane Katrina

	Depressive symptoms			PTSD symptoms		
		
	N	%	P	N	%	p
Overall	53	18		38	13	
						
Age			0.74			0.74
18–22	9	20		6	14	
>22–28	16	19		14	16	
>28–33	17	20		9	11	
>33	11	14		9	12	
						
Race			0.01			0.03
white	28	15		19	10	
black	25	30		18	22	
other	1	10		1	10	
						
Education			<0.01			0.04
< high school	2	10		1	5	
high school diploma	21	36		12	20	
some college/associates' degree	14	17		15	18	
college degree	9	11		4	5	
> college	7	19		6	16	
						
Parity			0.54			0.48
first child	19	16		13	11	
has other children	34	20		25	14	
						
Income			0.20			0.09
<$20000	12	20		11	18	
$20000–$60000	30	22		21	15	
>$60000	11	13		6	7	
						
Marital status			<0.01			0.1
married	26	14		20	11	
living with partner	11	20		7	13	
separated/divorced	4	50		2	25	
never married	12	32		9	24	

Most hurricane experiences were associated with worse mental health at two months postpartum (tables 3 and 4), and multivariable models indicated that these associations were not due to associations with the measured confounders. However, area of residence and evacuation were not associated with mental health overall. Results were similar in those pregnant during and after the hurricane, and race did not interact with the exposures to affect mental health. Adjustment for complications of pregnancy, delivery, and the infant did not substantially influence the results.

**Table 3:** Associations between aspects of exposure to Hurricane Katrina and postpartum depression in a cohort of postpartum southern Louisiana women

	Depressive symptoms				
	N	%	p	RR*	95% CI
Overall	53	18			
					
Home before the storm			0.84		
New Orleans area	38	19		0.85	(1.47, 0.50)
Baton Rouge area	15	18		1.00	
					
Substantial damage to house, property, for self and others			<0.01		
Yes	31	26		1.61	(0.98, 2.64)
No	22	13		1.00	
					
Injury to self or others			<0.01		
Yes	22	31		2.05	(1.27, 3.30)
No	31	14		1.00	
					
Perceived or experienced danger			<0.01		
Yes	30	28		1.92	(1.17, 3.15)
No	23	13		1.00	
					
Any 2 or more indicators of severe exposure			<0.01		
Yes	29	27		1.77	(1.08, 2.89)
No	24	13		1.00	
					
Left New Orleans before storm (asked only of New Orleans women)			0.29		
yes	34	19		1.43	(0.76, 2.70)
no	6	29		1.00	

*RR, relative risk; CI, confidence interval; Multivariable log-poisson models. All models are adjusted for age, race, parity, education, partnership status, and income.

**Table 4:** Associations between aspects of exposure to Hurricane Katrina and post-traumatic stress disorder in a cohort of postpartum southern Louisiana women

	PTSD symptoms*				
	N	%	p	RR**	95% CI
Overall	38	13			
					
Home before the storm			0.06		
New Orleans area	32	15		1.92	(0.81, 4.55)
Baton Rouge area	6	7		1.00	
					
Substantial damage to house, property, for self and others			<0.01		
Yes	24	20		2.06	(1.10, 3.88)
No	14	8		1.00	
					
Injury to self or others			<0.01		
Yes	17	24		2.46	(1.34, 4.54)
No	21	10		1.00	
					
Perceived or experienced danger			<0.01		
Yes	24	22		2.65	(1.35, 5.18)
No	14	8		1.00	
					
Any 2 or more indicators of severe exposure			<0.01		
Yes	26	24		3.68	(1.80, 7.52)
No	12	7		1.00	
					
Left New Orleans before storm (asked only of New Orleans women)			0.57		
yes	25	14		1.19	(0.46, 3.06)
no	4	18		1.00	

*PTSD, post-traumatic stress disorder; RR, relative risk; CI, confidence interval; Multivariable log-poisson models

**All models are adjusted for age, race, parity, education, partnership status, and income.

## Discussion

We are not aware of any other studies that address postpartum mental health after a natural disaster. Our study indicated that, like other people, postpartum women who experience natural disaster are more likely to develop mental health problems. We found, however, that a woman's own experience of the disaster, rather than generally living in a hard-hit area, was most predictive of mental health sequelae. Although we recruited the Baton Rouge group as a less-exposed comparison group, many women at this site also had a substantial experience of the storm. We also found that black and less educated women were more likely to be exposed to the worst of the storm and more likely to have mental health problems. However, black race and disaster experience did not interact to worsen mental health.

Strengths of the study include the use of standardized mental health instruments and systematic recruitment of an unselected population. In addition, we assessed the women's experience of the hurricane approximately two months prior to the first mental health measurements. Limitations include lack of clinical assessment. Generalizability is also a concern. Women who were most exposed to the storm may have been more or less likely to participate. The New Orleans sample was from the metro New Orleans area in general, not just New Orleans itself, so few experienced the most traumatic events (being rescued off a roof, spending time in the Superdome or Conference Center). Their experience may be more typical, however, for the entire affected region. Although selective participation and loss to follow-up may have influenced our overall prevalence, these problems affect all post-disaster studies. The time period of the study does not cover the immediate aftermath of the storm, nor does it include women who were permanently displaced. The hurricane occurred either during early pregnancy or prior to conception for these women, and analysis of those women who conceived prior to Katrina's landfall compared to those who conceived after landfall did not reveal major differences. This is not surprising; the period of severe stress lasted longer than several days or even several weeks. New Orleans remained flooded for three weeks, and most schools did not re-open until January 2006. Thus, mothers who became pregnant after landfall were still likely exposed to extensive hurricane-related stress. It is also possible that women with especially severe responses to the storm may have chosen to postpone childbearing. However, some research suggests that unplanned pregnancies are a consequence of natural disaster [37].

Other Katrina studies have generally found similar or higher levels of mental health problems. Desalvo et al., using the same instrument used in this study, found that 19% of the Tulane workforce met the criteria for PTSD six months after the disaster [35]. Unlike our study, they found that black race was protective, but similar to our study, they found experience of the storm to be correlated and higher education to be protective. The Hurricane Katrina Community Advisory Group study reported that 11% of prehurricane residents of the affected area had a serious anxiety-mood disorder, and 20% had a mild/moderate anxiety-mood disorder at interviews five to eight months after the storm. 16% met the criteria for PTSD based on the Trauma Screening Questionnaire [27]. They also found that those of low socioeconomic status (though not black race) were at increased risk for PTSD and mental illness, that differential exposure to the hurricane stressors did not explain the association between these demographic factors and mental health, and that there was not interaction between demographic variables and hurricane exposure. One year later, the prevalence of serious mental illness and PTSD had risen [38]. It is not clear why our sample should have a lower proportion of PTSD and depression than these surveys. Each of these surveys found a higher risk for mental health problems in women. The proportion of our postpartum study indicating at least a little nightmares (27%), irritability (60%), being easily startled (37%), or having upsetting memories (48%) was quite similar to Community Advisory Group Survey (25%, 52%, 38%, and 51%, respectively)[39]. This suggests that the women in our sample had low level symptoms to a similar degree to a broader sample, but that symptom levels did not rise to the level of pathology.

## Conclusion

Our study finds that many of the same forces that act on the general population also affect postpartum mental health, but that rates of mental health problems are not necessarily higher than for the general population. Postpartum women might even be at lower risk than the general population. Possibly women receive more social support and nurturing under difficult circumstances. Our results also suggest that members of the community who may be more vulnerable to the effects of disaster (for instance, those with less education) are likely to be vulnerable in part due to increased exposure to difficult experiences. This argues that planning to prevent severe exposures for the entire community is the strategy most likely to reduce mental health consequences.

## Abbreviations

PTSD: post-traumatic stress disorder; EPDS: Edinburgh Postpartum Depression Scale; PCL: Post-Traumatic Stress Checklist; RR: relative risk; CI: confidence interval.

## Competing interests

The authors declare they have no competing interests.

## Authors' contributions

EWH wrote the paper and directed the study. XX and PB contributed to study design, analysis, and paper writing. GP and KEH supervised study conduct and edited the paper. All authors approved the final version of the paper.

## Pre-publication history

The pre-publication history for this paper can be accessed here:


